# Final analysis of the randomised PEAK trial: overall survival and tumour responses during first-line treatment with mFOLFOX6 plus either panitumumab or bevacizumab in patients with metastatic colorectal carcinoma

**DOI:** 10.1007/s00384-017-2800-1

**Published:** 2017-04-19

**Authors:** Fernando Rivera, Meinolf Karthaus, J. Randolph Hecht, Isabel Sevilla, Frédéric Forget, Gianpiero Fasola, Jean-Luc Canon, Xuesong Guan, Gaston Demonty, Lee S. Schwartzberg

**Affiliations:** 1 Hospital Universitario Marqués de Valdecilla, Av. de Valdecilla, 39008 Santander, Spain; 2 Städtisches Klinikum München, Klinikum Neuperlach, Munich, Germany; 3 David Geffen School of Medicine, University of California Los Angeles, Los Angeles, CA USA; 4 Virgen de la Victoria University Hospital, Malaga, Spain; 5 Centre Hospitalier de l’Ardenne, Libramont, Belgium; 6 University Hospital Santa Maria della Misericordia, Udine, Italy; 7 Grand Hôpital de Charleroi, Charleroi, Belgium; 8 Amgen Inc., Biostatistics, Thousand Oaks, CA USA; 9 Medical Development – Oncology, Amgen (Europe) GmbH, Zug, Switzerland; 10 West Clinic, Memphis, TN USA

**Keywords:** Bevacizumab, First-line, Metastatic colorectal cancer, Overall survival, Panitumumab

## Abstract

**Purpose:**

To report planned final overall (OS) and progression-free survival (PFS) analyses from the phase II PEAK trial (NCT00819780).

**Methods:**

Patients with previously untreated, *KRAS* exon 2 wild-type (WT) metastatic colorectal cancer (mCRC) were randomised to mFOLFOX6 plus panitumumab or bevacizumab. The primary endpoint was PFS; secondary endpoints included OS, objective response rate, duration of response (DoR), time to response, resection and safety. Treatment effect by tumour *RAS* status was a prespecified objective. Exploratory analyses included early tumour shrinkage (ETS) and depth of response (DpR).

**Results:**

One hundred seventy patients had *RAS* WT and 156 had *RAS* WT/*BRAF* WT mCRC. Median PFS was longer for panitumumab versus bevacizumab in the *RAS* WT (12.8 vs 10.1 months; hazard ratio (HR) = 0.68 [95% confidence intervals (CI) = 0.48–0.96]; *p =* 0.029) and *RAS* WT/*BRAF* WT (13.1 vs 10.1 months; HR = 0.61 [95% CI = 0.42–0.88]; *p =* 0.0075) populations. Median OS (68% OS events) for panitumumab versus bevacizumab was 36.9 versus 28.9 months (HR = 0.76 [95% CI = 0.53–1.11]; *p =* 0.15) and 41.3 versus 28.9 months (HR = 0.70 [95% CI = 0.48–1.04]; *p =* 0.08), in the *RAS* WT and *RAS* WT/*BRAF* WT populations, respectively. Median DoR (11.4 vs 9.0 months; HR = 0.59 [95% CI = 0.39–0.88]; *p =* 0.011) and DpR (65.0 vs 46.3%; *p =* 0.0018) were improved in the panitumumab group. More panitumumab patients experienced ≥30% ETS at week 8 (64 vs 45%; *p =* 0.052); ETS was associated with improved PFS/OS. No new safety signals occurred.

**Conclusions:**

First-line panitumumab + mFOLFOX6 increases PFS versus bevacizumab + mFOLFOX6 in patients with *RAS* WT mCRC.

**Electronic supplementary material:**

The online version of this article (doi:10.1007/s00384-017-2800-1) contains supplementary material, which is available to authorized users.

## Introduction

First-line treatments for patients with metastatic colorectal cancer (mCRC) comprise chemotherapy combined with an epidermal growth factor receptor inhibitor (EGFRI), panitumumab or cetuximab, or the vascular endothelial growth factor inhibitor (VEGFI) bevacizumab [1]. Confirmation of wild-type (WT) tumour *RAS* status is essential ahead of prescribing EGFRIs [1, 2], the importance of evaluating tumour *RAS* status ahead of bevacizumab treatment is less clear [3, 4]. In the EU, panitumumab is indicated in combination with FOLFOX or FOLFIRI for the first-line treatment of patients with *RAS* WT (no mutations in exons 2, 3 and 4 of *KRAS* and *NRAS*) mCRC [5]. In the USA, panitumumab is indicated in *KRAS* (exon 2) WT mCRC combined with FOLFOX for first-line treatment, and as monotherapy following disease progression after prior treatment with fluoropyrimidine, oxaliplatin and irinotecan-containing chemotherapy [6]. Bevacizumab is also indicated in the EU for the treatment of patients with mCRC in combination with fluoropyrimidine-based chemotherapy [7] and in the USA with intravenous 5-fluorouracil-based chemotherapy for first- or second-line mCRC treatment and with fluoropyrimidine-irinotecan- or fluoropyrimidine-oxaliplatin-based chemotherapy for second-line treatment after progression (PD) on a first-line bevacizumab-containing regimen [8].

PEAK (Panitumumab Efficacy in combination with mFOLFOX6 Against bevacizumab plus mFOLFOX6 in mCRC subjects with *KRAS* WT tumours) is a phase II, randomised study evaluating the efficacy and safety of first-line panitumumab + modified FOLFOX6 (mFOLFOX6) versus bevacizumab + mFOLFOX6 in patients with *KRAS* exon 2 WT mCRC [9]. The primary objective was to assess progression-free survival (PFS) in the *KRAS* exon 2 WT population; a prespecified secondary objective was to assess PFS and overall survival (OS) in patients with *RAS* WT mCRC. In the primary analysis of PEAK, first-line panitumumab + mFOLFOX6 was associated with numerically longer OS than bevacizumab + mFOLFOX6 in patients with *RAS* WT mCRC (41% OS events; median OS = 41.3 vs 28.9 months; hazard ratio (HR) = 0.63 [95% confidence intervals (CI) = 0.39–1.02]; *p =* 0.058) and significantly longer PFS (median PFS = 13.0 vs 9.5 months; HR = 0.65 [95% CI = 0.44–0.96]; *p =* 0.029) [9]. Overall response rates (ORR) by Response Evaluation Criteria In Solid Tumours (RECIST) were similar between treatments (63.6 vs 60.5%, for panitumumab vs bevacizumab groups, respectively) [9]. RECIST, however, does not fully take into account timing, depth (DpR) and duration of response (DoR), all of which may influence long-term outcomes [10, 11].

In addition to *RAS* mutations, other biomarkers have potential predictive and prognostic importance in mCRC. *BRAF* mutation status has emerged as a strong prognostic marker [12] but there is a lack of evidence to support best approaches to treatment for *BRAF* mutant (MT) mCRC [1, 2]. Clinical trials may, therefore, be the best option for some patients. Alternatively, a small population sub-analysis from the TRIBE trial suggested a potential OS benefit for patients with *BRAF* mutations receiving bevacizumab + FOLFOXIRI (*n =* 16) versus bevacizumab + FOLFIRI (*n =* 12) [13], and so, this combination may be an option for patients with good performance status.

Here, we report updated OS and PFS from a planned final analysis of PEAK data (*RAS* WT and *RAS* WT/*BRAF* WT populations). Exploratory analyses of tumour assessments beyond RECIST were also performed, focussing on tumour dynamics (early tumour shrinkage (ETS), depth of response (DpR) and changes in tumour load over time (*RAS* WT population only)).

## Material and methods

### Study design and patients

PEAK (NCT00819780) was a phase II, randomised (1:1), open-label first-line study in patients with previously untreated *KRAS* exon 2 WT mCRC. Treatment continued until PD, unacceptable toxicity, death, consent withdrawal, or investigator decision. The trial was conducted in compliance with the Declaration of Helsinki. The study protocol was approved by an independent ethics committee at each participating study centre. All patients provided informed consent before any study procedures were performed.

Key eligibility criteria for PEAK (primary analysis) included age ≥18 years; an Eastern Cooperative Oncology Group performance score of 0/1; histologically/cytologically confirmed mCRC with unresectable metastatic disease; *KRAS* exon 2 (codons 12 and 13) WT tumour status; one unidimensionally measurable lesion of ≥20 mm (per modified RECIST guidelines); and no prior chemotherapy, anti-EGFR therapy or bevacizumab therapy for mCRC.

### Extended *RAS* analyses and *BRAF* mutation analyses

An extended *RAS* analysis was prespecified in PEAK [9]. In brief, banked tumour samples for patients with *KRAS* exon 2 WT tumours were tested for prespecified mutations in *NRAS* exon 2 (codons 12 and 13), and *KRAS* and *NRAS* exons 3 (codons 59 and 61) and 4 (codons 117 and 146). Mutations were also assessed in exon 15 (codon 600) of *BRAF* (exploratory analysis). Mutation status was assessed using bidirectional Sanger sequencing and WAVE-based SURVEYOR^®^ scan kits (Transgenomic; Omaha, NE, USA), as reported previously [12].

### Efficacy assessments

Endpoints reported here include PFS, OS, ORR, DoR, time to response (TTR), resection rates, DpR, change in tumour load versus baseline (over time), and ETS. Tumour response was assessed by the investigator every 8 weeks (±7 days) using modified RECIST (version 1.0; there was no independent review). ETS was defined as the proportion of patients with tumour shrinkage at week 8; two cutoff values were used, ≥30 and ≥20%. Tumour shrinkage was defined as the change in sum of the longest diameters (SLD) of all target lesions. DpR was defined for each patient as the percentage of tumour shrinkage at nadir or progression. DpR has a positive value for tumour reduction, a negative value for tumour growth and is zero for no change. If shrinkage occurred during treatment, then DpR was the greatest percentage tumour shrinkage observed at nadir versus baseline. In all other cases, DpR was defined as the percentage of tumour change at progression. If progression did not occur, DpR was not defined as PFS could not be defined. Changes in tumour load were calculated as mean (95% CI) percentage change from baseline in the SLD of all target lesions over time.

### Safety assessments

Patients were followed-up for safety until 30 days after the last dose of study drug. Adverse events (AEs) were graded using Common Terminology Criteria for Adverse Events (version 3.0), with modifications for specific skin- and nail-related toxicities.

### Statistical analyses

The primary endpoint in PEAK was PFS; secondary endpoints included OS, ORR, DoR, TTR, resection rates, and safety (all reported here for the *RAS* WT population). Six-month PFS and 2-year OS rates by resection status were calculated by treatment (exploratory analysis). Exploratory analyses of DpR, changes in tumour load versus baseline and ETS, were performed for the *RAS* WT population; median PFS and OS were calculated for patients with/without ≥30 and ≥20% ETS, by treatment and overall. Top-line efficacy results (PFS, OS and ORR) were also reported for the *RAS* WT*/BRAF* WT and *RAS* WT/*BRAF* MT populations.

The treatment effect HR for panitumumab relative to bevacizumab combined with mFOLFOX6 and the associated 95% CIs were estimated from a Cox model stratified by previous adjuvant oxaliplatin therapy. Kaplan-Meier curves were generated for all time-to-event endpoints.

The primary analysis of PEAK was performed in 2012 when the target of ~168 PFS events was achieved [9]. This PFS event goal was expected to be achieved with recruitment of approximately 280 patients. The current final analysis was planned to occur 36 months after the last patient enrolled. No formal hypothesis testing was planned; all reported *p* values are descriptive.

The prespecified extended *RAS* analysis included all intent-to-treat (ITT) patients with *RAS* data available. The extended *RAS* statistical analysis plan was developed before the data analysis was conducted and no biomarker testing results were available to the study team before plan approval.

## Results

### Patients

**Fig. 1:**
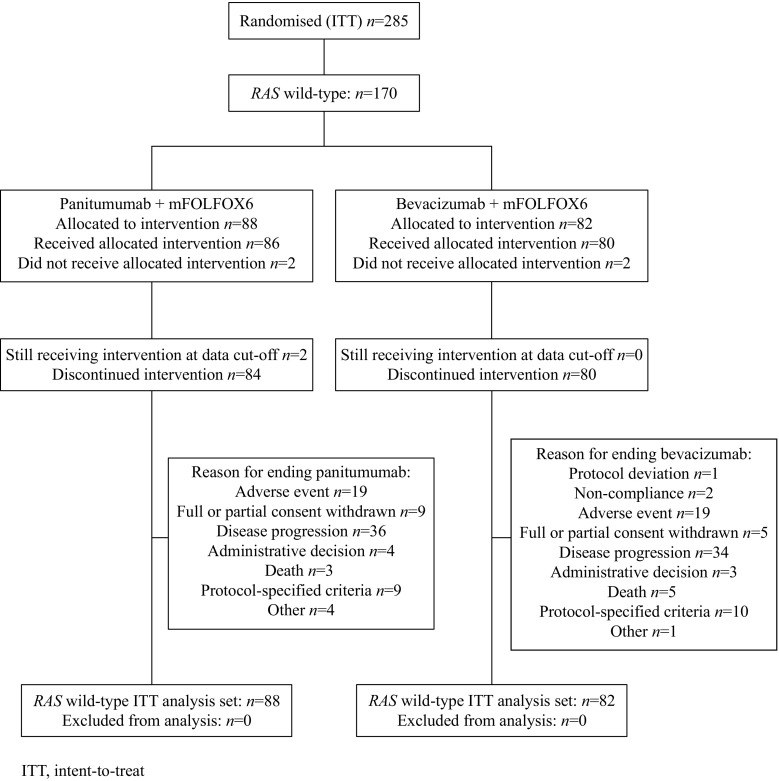
PEAK study CONSORT diagram (data cutoff 11 February 2015)

Between April 2009 and December 2011, 285 patients with *KRAS* exon 2 WT mCRC were enrolled from 60 sites; 142 patients were randomly assigned to panitumumab + mFOLFOX6 and 143 to bevacizumab + mFOLFOX6 (primary analysis ITT set [Fig. 1]). Overall, 278 patients received treatment. Samples from 250 patients in the ITT population (88%) underwent extended *RAS* analysis, with 233 (82%) having a result. Twelve patients were identified as having *KRAS* exon 2 mutations upon additional testing, giving a *KRAS* exon 2 WT population of 221. Among these 221 patients, 170 (77%) had *RAS* WT mCRC. *BRAF* mutations were found in tumours from 14 patients (panitumumab + mFOLFOX6 *n =* 11; bevacizumab + mFOLFOX6 *n =* 3), all of whom had *RAS* WT mCRC.

Baseline demographics and disease characteristics were generally similar between treatments in both the *RAS* WT and *RAS* WT*/BRAF* WT populations (Table 1). The mean (standard deviation) actual follow-up time was 134.1 (73.8) weeks in the panitumumab arm and 115.7 (71.0) weeks in the bevacizumab arm. Overall, the median duration of panitumumab treatment was 14.0 cycles in both the *RAS* WT and *RAS* WT/*BRAF* WT populations. The median duration of bevacizumab treatment was 11.0 and 11.5 cycles in the *RAS* WT and *RAS* WT/*BRAF* WT populations, respectively.

**Table 1:** Baseline demographics and disease characteristics

	*RAS* wild-type		*RAS* wild-type/*BRAF* wild-type	
	Panitumumab + mFOLFOX6 (*n =* 88)	Bevacizumab + mFOLFOX6 (*n =* 82)	Panitumumab + mFOLFOX6 (*n =* 77)	Bevacizumab + mFOLFOX6 (*n =* 79)
Male sex, *n* (%)	58 (66)	56 (68)	50 (65)	55 (70)
Age, years – median (range)	62 (23–82)	60 (39–82)	62 (23–82)	60 (39–82)
ECOG PS, *n* (%)				
0 or 1	88 (100)	81 (99)	77 (100)	78 (99)
Missing	0 (0)	1 (1)	0 (0)	1 (1)
Primary tumour diagnosis, *n* (%)				
Colon	64 (73)	57 (70)	53 (69)	54 (68)
Rectum	24 (27)	25 (30)	24 (31)	25 (32)
Primary tumour location, *n* (%)				
Left	53 (60)	54 (66)	52 (68)	53 (67)
Right	22 (25)	14 (17)	13 (17)	13 (16)
Unknown/unavailable	13 (15)	14 (17)	12 (16)	13 (16)
Sites of metastases, *n* (%)				
Liver only	23 (26)	22 (27)	21 (27)	22 (28)
Liver + other	43 (49)	34 (41)	37 (48)	33 (42)
Other only	22 (25)	26 (32)	19 (25)	23 (29)
Number of metastatic sites, median (range)	2 (1–5)	2 (0–4)	2 (1–5)	2 (0–4)
Sum of longest diameters of all target lesions, mm – mean (standard deviation)^a^	129.0 (117.2)	109.1 (84.5)	131.6 (123.5)	110.2 (85.2)
CEA levels, μg/L – median (range)	12.8 (0–8543)	15.2 (1–4889)	12.6 (0–8453)	15.6 (1–4889)

^a^Sum of longest diameters of target lesions was missing/unknown for one patient in the bevacizumab group

*CEA* carcinoembryonic antigen, *ECOG PS* Eastern Cooperative Oncology Group performance status

### Efficacy

**Fig. 2:**
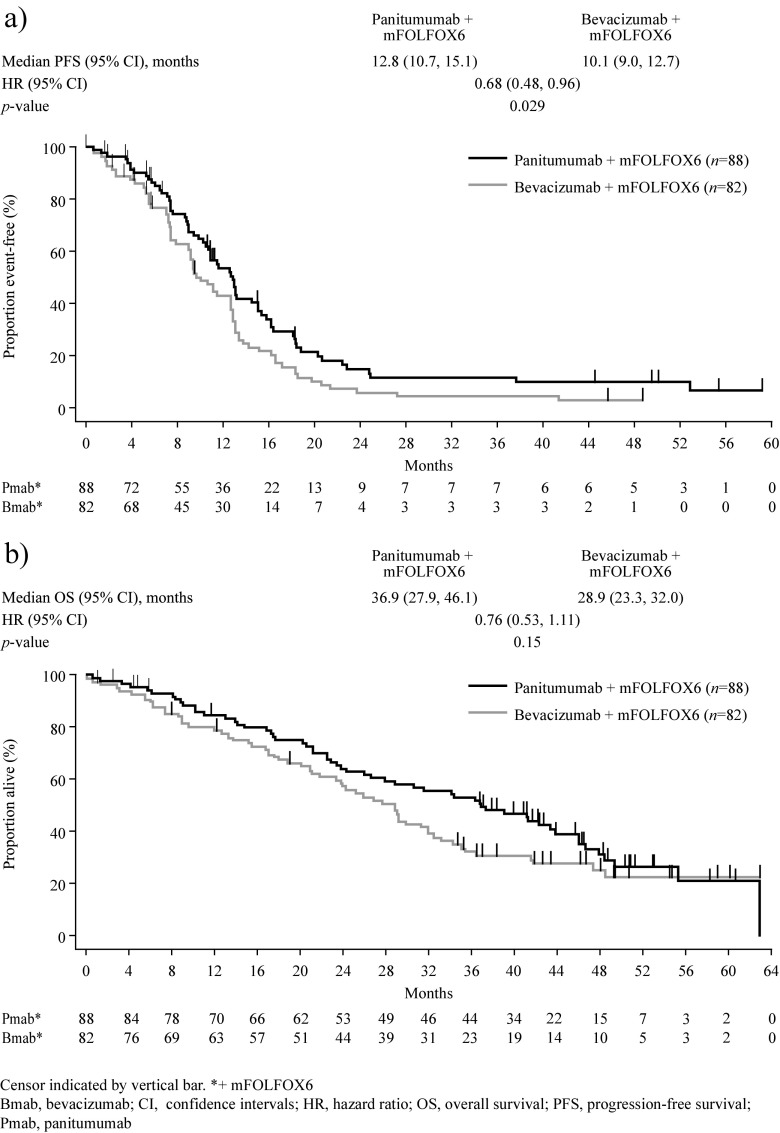
Kaplan-Meier estimates of **a** Progression-free survival and **b** Overall survival (*RAS* wild-type population)

At the time of analysis, 73 versus 85% of patients with *RAS* WT mCRC receiving panitumumab + mFOLFOX6 versus bevacizumab + mFOLFOX6, respectively, had PFS events (Table 2). In the *RAS* WT population, PFS was longer in the panitumumab versus bevacizumab group (median = 12.8 vs 10.1 months; HR = 0.68 [95% CI = 0.48–0.96]; *p =* 0.0292) (Fig. 2a).

**Table 2:** Summary of progression-free survival, overall survival and objective response results

	*RAS* wild-type		*RAS* wild-type/*BRAF* wild-type	
	Panitumumab + mFOLFOX6 (*n =* 88)	Bevacizumab + mFOLFOX6 (*n =* 82)	Panitumumab + mFOLFOX6 (*n =* 77)	Bevacizumab + mFOLFOX6 (*n =* 79)
Progression-free survival				
Patients with event, *n* (%)	64 (73)	70 (85)	55 (71)	67 (85)
Median, months (95% CI)	12.8 (10.7, 15.1)	10.1 (9.0, 12.7)	13.1 (11.6, 16.2)	10.1 (9.0, 12.7)
HR (95% CI)	0.68 (0.48, 0.96)		0.61 (0.42, 0.88)	
*p* value*	0.029		0.0075	
Overall survival				
Patients with event, *n* (%)	57 (65)	58 (71)	48 (62)	55 (70)
Median, months (95% CI)	36.9 (27.9, 46.1)	28.9 (23.3, 32.0)	41.3 (31.6, 46.7)	28.9 (23.9, 33.1)
HR (95% CI)	0.76 (0.53, 1.11)		0.70 (0.48, 1.04)	
*p* value*	0.15		0.08	
Subsequent therapy				
Anti-EGFR mAb, *n* (%)	28 (32)	41 (50)	27 (35)	40 (51)
Anti-VEGF mAb, *n* (%)	44 (50)	31 (38)	39 (51)	30 (38)
Objective response^†^				
Responders,^‡^ *n*	57	49	50	48
ORR,^‡^ % (95% CI)	65 (54, 75)	60 (49, 71)	65 (53, 75)	62 (50, 72)
Difference in rates, % (95% CI)	4.3 (−10.9, 19.3)		3.4 (−12.5, 19.0)	
Odds ratio^¶^ (95% CI)	1.12 (0.56, 2.22)		1.11 (0.54, 2.27)	
*p* value^§^	0.86		0.90	

*CI* confidence interval, *EGFR* epidermal growth factor receptor, *HR* hazard ratio, *mAb* monoclonal antibody, *ORR* objective response rate, *RECIST* Response Evaluation Criteria In Solid Tumours, *VEGF* vascular endothelial growth factor, *WT* wild type

*From stratified Cox model

^†^For the objective response analysis, *n =* 81 for the *RAS* WT bevacizumab group and *n =* 78 for the *RAS* WT/*BRAF* WT bevacizumab group

^‡^As assessed by RECIST

^¶^Defined as the odds of having an objective response in the panitumumab + mFOLFOX6 arm relative to the odds in the bevacizumab + mFOLFOX6 arm

^§^From stratified exact test

Mortality rates in the *RAS* WT population at the time of analysis were 65 versus 71% for the panitumumab + mFOLFOX6 versus bevacizumab + mFOLFOX6 groups, respectively (Table 2). In the *RAS* WT population, median OS was 36.9 versus 28.9 months in the panitumumab versus bevacizumab groups, respectively (HR = 0.76 (95% CI = 0.53–1.11)); *p =* 0.15) (Fig. 2b). Although some patient subpopulations were small, the trend (not statistically significant) towards improved OS in the panitumumab group was generally observed across subpopulations defined by baseline covariates (Online Resource Fig. A1). Similar proportions of patients in the panitumumab and bevacizumab groups subsequently crossed over to anti-VEGF and anti-EGFR therapies, respectively (Table 2).

Overall, 169 patients with *RAS* WT mCRC were included in the ORR analysis; ORRs were similar in the panitumumab + mFOLFOX6 and bevacizumab + mFOLFOX6 arms (65 vs 60%, respectively; odds ratio = 1.12 (95% CI = 0.56–2.22); *p =* 0.86) (Table 2).

Similar observations for PFS (median = 13.1 vs 10.1 months; HR = 0.61 (95% CI = 0.42–0.88); *p =* 0.0075), OS (median = 41.3 vs 28.9 months; HR = 0.70 (95% CI = 0.48–1.04); *p =* 0.08) and ORR (65 vs 62%) were found for the panitumumab versus bevacizumab groups, respectively, in the *RAS* WT*/BRAF* WT population (Table 2; Online Resource Fig. A2). The efficacy of panitumumab and bevacizumab in patients with *RAS* WT/*BRAF* MT disease could not be determined due to the small number of patients in this subpopulation (*n =* 11 and *n =* 3, respectively; Online Resource Table A1).

In patients with *RAS* WT mCRC, median TTR was numerically shorter, median DoR longer and median DpR greater in the panitumumab + mFOLFOX6 versus bevacizumab + mFOLFOX6 group (Table 3). Resection rates and outcomes following resection were similar between treatments for the *RAS* WT population, although sample sizes were small for this analysis (Table 3).

**Table 3:** Summary of response- and resection-related efficacy results (*RAS* wild-type population)

	Panitumumab + mFOLFOX6 (*n =* 88)	Bevacizumab + mFOLFOX6 (*n =* 82)*
Median DoR, months (95% CI)	11.4 (10.0, 16.3)	9.0 (7.6, 9.5)
HR^†^ (95% CI)	0.59 (0.39, 0.88)	
*p* value^‡^	0.011	
Median TTR, months (95% CI)	2.3 (1.9, 3.7)	3.8 (2.1, 5.7)
HR^†^ (95% CI)	1.19 (0.81, 1.74)	
*p* value^‡^	0.37	
Median DpR, % (Q1, Q3)	65.0 (45.7, 89.5)	46.3 (29.5, 63.3)
*p* value^¶^	0.0018	
Any resection, *n* (%)	12 (14)	9 (11)
Liver only^§^	9 (75)	6 (67)
Complete resection	9 (10)	7 (9)
Liver only^^^	7 (78)	6 (86)
Time to resection, months – median (range)	5.1 (3–19)	4.4 (3–12)
Progression-free at 6 months, *n* (%)		
Patients with resection	8/12 (67)	4/9 (44)
Patients without resection	45/76 (59)	48/73 (66)
Alive at 2 years, *n* (%)		
Patients with resection	8/12 (67)	8/9 (89)
Patients without resection	18/76 (24)	12/73 (16)

*CI* confidence interval, *DoR* duration of response, *DpR* depth of response, *HR* hazard ratio, *TTR* time to response

*For DoR, TTR and DpR analyses, *n =* 81 for the bevacizumab group

^†^HRs are presented as panitumumab + mFOLFOX6 : bevacizumab + mFOLFOX6. A value <1.0 indicates a lower average event rate and longer time to event for panitumumab + mFOLFOX6 relative to bevacizumab + mFOLFOX6

^‡^For treatment effect

^¶^ *p* value from Wilcoxon test

^§^Percentage is calculated using the number of patients who had resection as the denominator

^^^Percentage is calculated using the number of patients who had complete resection as the denominator

**Fig. 3:**
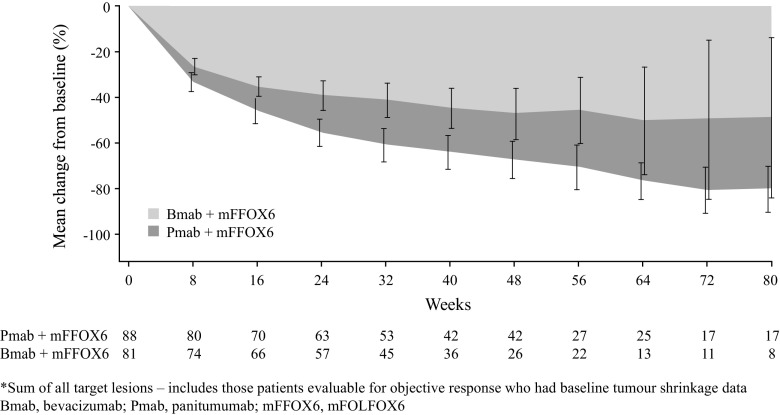
Mean (95% confidence interval) percentage change from baseline in tumour load (sum of all target lesions) over time (*RAS* wild-type population)^*^

The mean percentage change from baseline in tumour load in the *RAS* WT population appeared to favour panitumumab at all measured time points, although the CIs were wide (Fig. 3). Figure 3 shows tumour data at each scheduled visit; following PD, patients were only followed up for survival and no further CT scans were analysed.

Overall, 154 patients with *RAS* WT mCRC had tumour shrinkage data available at baseline and week 8. Compared with the bevacizumab + mFOLFOX6 arm, more patients in the panitumumab + mFOLFOX6 group experienced ≥30% ETS (Table 4). Numerically, more patients in this group also had ≥20% ETS. For those achieving ≥30 or ≥20% ETS, numerically longer PFS and OS were seen in the panitumumab versus bevacizumab arm (Table 4; Online Resource Fig. A3). ETS (≥30 or ≥20%) was associated with numerically longer PFS and OS, irrespective of treatment received (Online Resource Table A2). When treatment arms were combined, achievement of ≥30 versus <30% ETS was associated with longer PFS (median = 12.9 vs 9.8 months; HR = 0.60 (95% CI = 0.42–0.87); *p =* 0.0065) and OS (median = 43.4 vs 24.3 months; HR = 0.44 (95% CI = 0.30–0.65); *p* < 0.0001). Similar results were seen when combined data were analysed using the ≥20% ETS cutoff (median PFS = 13.0 vs 9.5 months; HR = 0.55 [95% CI = 0.37–0.81]; *p =* 0.0029; median OS = 39.1 vs 21.2 months; HR = 0.39 [95% CI = 0.26–0.59]; *p* < 0.0001).

**Table 4:** Progression-free and overall survival outcomes by tumour shrinkage at week 8 (*RAS* wild-type population – between treatment comparisons)

	Tumour shrinkage at week 8			
	Panitumumab + mFOLFOX6	Bevacizumab + mFOLFOX6	Panitumumab + mFOLFOX6	Bevacizumab + mFOLFOX6
	<30%		≥30%	
Patients with shrinkage, *n* (%)	29 (36)	41 (55)	51 (64)	33 (45)
Odds ratio* (95% CI)	1.99 (0.99, 4.10)			
*p* value	0.052			
Median PFS, months (95% CI)	11.6 (7.5, 15.4)	9.7 (7.5, 12.9)	13.0 (10.9, 18.1)	11.1 (9.0, 16.6)
HR (95% CI)	0.79 (0.45, 1.38)		0.74 (0.45, 1.23)	
*p* value	0.40		0.24	
Median OS, months (95% CI)	34.2 (17.5, 42.3)	23.9 (20.1, 29.0)	43.8 (36.4, 63.0)	35.1 (29.9, NE)
HR (95% CI)	0.75 (0.43, 1.31)		0.77 (0.42, 1.42)	
*p* value	0.31		0.41	
	<20%		≥20%	
Patients with shrinkage, *n* (%)	20 (25)	28 (38)	60 (75)	46 (62)
Odds ratio* (95% CI)	1.67 (0.78, 3.58)			
*p* value	0.21			
Median PFS, months (95% CI)	9.8 (4.2, 15.4)	9.5 (7.4, 12.7)	13.1 (10.9, 16.2)	11.3 (9.2, 13.6)
HR (95% CI)	0.99 (0.50, 1.95)		0.70 (0.45, 1.08)	
*p* value	0.97		0.11	
Median OS, months (95% CI)	21.2 (14.1, 41.2)	21.8 (15.3, 28.9)	43.4 (36.4, 55.4)	32.5 (27.7, 47.4)
HR (95% CI)	0.81 (0.42, 1.57)		0.73 (0.44, 1.19)	
*p* value	0.53		0.21	

*Odds ratio is defined as the odds of having ≥30 or ≥20% tumour shrinkage in the panitumumab + mFOLFOX6 arm relative to the odds in the bevacizumab + mFOLFOX6 arm

*CI* confidence intervals, *HR* hazard ratio, *NE* not evaluable, *OS* overall survival, *PFS* progression-free survival

### Safety

In the *RAS* WT safety analysis population, similar proportions of patients in the panitumumab and bevacizumab groups experienced AEs (100 vs 100%), worst grade 3 or 4 AEs (90 vs 73%), serious AEs (43 vs 40%), fatal AEs (5 vs 9%) and discontinuations (any drug) due to AEs (29 vs 30%) (Online Resource Table A3). Overall, 20% of patients discontinued panitumumab and 25% discontinued bevacizumab treatment due to an AE. In the panitumumab arm, four fatal AEs (5%) were reported: cardiac arrest, pneumonia aspiration, respiratory failure and sepsis (*n =* 1 for each). In the bevacizumab arm, seven fatal AEs (9%) were reported: cardiac arrest, sepsis, cardio-respiratory arrest, intestinal obstruction, intestinal perforation, septic shock and small intestinal perforation (*n =* 1 for each). Overall, 36 versus 3% of patients in the panitumumab versus bevacizumab groups had grade ≥3 skin/subcutaneous tissue disorders; 5 versus 18% of patients in these groups, respectively, had grade ≥3 vascular disorders. There was a ≥5% difference in incidence rate between treatment groups for the following grade ≥3 AEs (panitumumab vs bevacizumab): rash (15 vs 0%), hypomagnesaemia (8 vs 0%), stomatitis (7 vs 0%), decreased appetite (6 vs 1%), dehydration (6 vs 1%), acne (5 vs 0%), dermatitis acneiform (5 vs 0%), deep vein thrombosis (2 vs 8%), and hypertension (0 vs 8%) (Online Resource Table A3).

## Discussion

The efficacy results from this final analysis of PEAK were consistent with the results of the primary analysis of this trial [9]. Patients with *RAS* WT tumours receiving panitumumab + mFOLFOX6 had longer PFS (12.8 vs 10.1 months, *p =* 0.029) and DoR (11.4 vs 9.0 months, *p =* 0.011) compared with those receiving bevacizumab + mFOLFOX6. ORR (65 vs 60%, *p =* 0.86) and resection rates (14 vs 11%) were similar between treatments. Exploratory analyses of DpR (median 65 vs 46%, *p =* 0.0018) and ETS (≥30% ETS 64 vs 45%, *p =* 0.052) suggested improvements in these measures for the panitumumab versus bevacizumab group. In line with previous reports [10, 11, 14, 15], ETS appeared to be associated with improved PFS and OS, irrespective of first-line treatment received. A limitation to this study is that radiologic assessment was performed by the investigator and no independent radiologic review was performed.

In the *RAS* WT population, median OS was 8 months longer in the panitumumab group (36.9 vs 28.9 months; HR = 0.76 [95% CI = 0.53–1.11]; *p =* 0.15). Median OS was further extended in patients with *RAS* WT*/BRAF* WT tumours receiving panitumumab (41.3 months), but remained the same as for the *RAS* WT population in *RAS* WT*/BRAF* WT patients receiving bevacizumab (28.9 months). This is of note because patients with *BRAF* MT mCRC have a poor prognosis [12] and there is a small imbalance in the number of patients with *BRAF* MT tumours in this study (panitumumab arm *n =* 11; bevacizumab arm *n =* 3). However, the primary endpoint in PEAK was not OS and this trial was not powered to detect an OS increase, which may explain why the observed difference between arms was not statistically significant. The overall findings from PEAK nonetheless suggest an OS benefit for EGFRIs + chemotherapy versus bevacizumab + chemotherapy, which is consistent with results from FIRE-3 [16] and a congress presentation of limited data for the FOLFOX arm of the CALGB/SWOG 80405 study (full publication not yet available) [17]. This is also in line with results from two recent meta-analyses of these trials, which included primary analysis data from PEAK. Both meta-analyses demonstrated significantly improved ORR and OS with first-line anti-EGFR compared with anti-VEGF therapy in patients with *RAS* WT mCRC [18, 19]. The HR (95% CI) for OS was 0.77 (0.63–0.95) in favour of the EGFRI in both analyses. Similar results were also observed in a meta-analysis including seven first-line trials comparing panitumumab or cetuximab plus chemotherapy with chemotherapy alone or chemotherapy plus bevacizumab [20].

The median OS of 41.3 months achieved in patients with *RAS* WT*/BRAF* WT mCRC receiving panitumumab in PEAK, compares well with other data for first-line biologics combined with doublet chemotherapy [12, 13]. Data have recently been reported for triplet chemotherapy combined with biologics in several studies including TRIBE [13, 21] (bevacizumab), TRIP [22] and VOLFI [23] (panitumumab) and MACBETH [24] (cetuximab), indicating that FOLFOXIRI may offer superior efficacy over doublet chemotherapy in the first-line setting. However, improved outcomes are achieved at the expense of increased grade 3/4 toxicity, including neutropenia, diarrhoea, stomatitis and neurotoxicity, which may limit the use of such regimens in clinical practice.

Although the OS results from PEAK are now more mature (68 and 66% deaths in the *RAS* WT and *RAS* WT*/BRAF* WT populations, respectively), these results need to be considered in context of the fact that PEAK is a phase II trial. There are several possible reasons for the prolonged OS observed. For example, the improved DoR in the panitumumab + mFOLFOX6 arm could be linked with the improved PFS and numerically improved OS seen in this arm. Likewise, the improved DpR, ETS and tumour load changes in the panitumumab versus bevacizumab arm could also play a role in improving long-term outcomes, as has been suggested in other reports [11, 25]. It is unlikely that the numeric, between-treatment OS differences were driven by resection, as resection rates in patients with *RAS* WT tumours were balanced between groups. It is also unlikely to be driven by differences in tumour side as the proportion of patients with left- and right-sided disease was well-balanced between treatment groups. As was suggested in FIRE-3 [26], the impact of treatment sequence could also be important, given that the proportions of patients switching biologic approach were balanced between arms (i.e. EGFRI to VEGFI or VEGFI to EGFRI). Preclinical data suggest that first-line EGFRI treatment may sensitise tumours to subsequent therapy, whereas first-line VEGFI may desensitise tumours [27–29]. This provides a biological rationale for first-line EGFRI therapy followed by second-line VEGFI therapy.

As well as comparative efficacy, safety/tolerability and cost-effectiveness of the available treatments need to be considered when choosing first-line therapy for patients with *RAS* WT mCRC. Updated data for the *RAS* WT population in PEAK suggest that the safety profiles of the two treatments are consistent with previously reported studies and no new toxicities or safety signals were identified. The main tolerability differences were due to known class effects of these treatments, e.g. skin rash/toxicities and hypomagnesaemia with the EGFRI and deep vein thrombosis and hypertension with bevacizumab. Discontinuation rates due to AEs were similar between arms (29 vs 30%, respectively).

Tumour biomarker status is increasingly being used to guide treatment decisions in mCRC and is a rapidly developing area of research. By targeting drugs only to those patients most likely to benefit, predictive biomarkers may improve clinical outcomes and also potentially reduce overall treatment costs. The availability of banked tumour samples from clinical studies is important to permit prospective, prospective-retrospective or retrospective analyses to be performed to test new biomarker hypotheses [30]. Analysis of data from the phase III PRIME study of panitumumab + FOLFOX4 confirmed the importance of *RAS* testing beyond *KRAS* in patients with mCRC and was possible due to the availability of *RAS* data for 90% of the *KRAS* WT tumour samples [12]. The *RAS* ascertainment rate in the PEAK study was also high (82%) in comparison to currently available data from other first-line mCRC studies comparing EGFRIs versus VEGFIs (FIRE-3 = 69% [16]; CALGB 80405 = 55% [17]), highlighting the importance of including the availability of paraffin-embedded tumour tissue for biomarker testing as a requirement for study entry. Mandating baseline biopsy and planning the conduct of prospective/prespecified biomarker analyses in clinical trials, can improve the robustness of the study data and future analyses. While tumour *RAS* status is undoubtedly a valuable predictive marker for response to EGFRIs, it remains important to investigate the roles of other potential biomarkers that could improve patient selection (e.g. epiregulin/amphiregulin, PIK3CA, PTEN). Data from the PRIME study indicated that *BRAF* mutations are linked with poor outcomes irrespective of treatment received [12]. *RAS* mutations do not appear to be prognostic for patients with mCRC.

In summary, ORR by RECIST is the accepted standard for assessing response in solid tumours, but has limitations in terms of assessing dynamic tumour burden changes. Although ORRs were similar between treatments in PEAK, tumour responses in the panitumumab arm appeared to occur earlier, last longer and be deeper compared with those occurring in the bevacizumab arm in these exploratory analyses. More patients receiving panitumumab + mFOLFOX6 versus bevacizumab + mFOLFOX6 had ≥30% ETS; ETS of ≥30 or ≥20% appeared to be associated with improved PFS and OS, irrespective of treatment received. PFS was also significantly improved for patients with *RAS* WT mCRC receiving panitumumab + mFOLFOX6 versus bevacizumab + mFOLFOX6. For panitumumab versus bevacizumab, median OS was 8.0 months longer in the *RAS* WT (36.9 vs 28.9 months) and 12.4 months longer in the *RAS* WT*/BRAF* WT (41.3 vs 28.9 months) populations, respectively (neither difference being statistically significant). Based on these data, panitumumab + mFOLFOX6 can be considered an effective first-line treatment for patients with *RAS* WT mCRC. A phase III trial is warranted to more fully evaluate any potential differences between panitumumab and bevacizumab treatment in the first-line setting.

## Electronic supplementary material

ESM 1 (DOCX 1211 kb)
