# Supplementary material for: Final analysis of the randomised PEAK trial: overall survival and tumour responses during first-line treatment with mFOLFOX6 plus either panitumumab or bevacizumab in patients with metastatic colorectal carcinoma
Source: Int J Colorectal Dis. 2017 Apr 19;32(8):1179–90. doi: 10.1007/s00384-017-2800-1 (PMC5522523; doi:10.1007/s00384-017-2800-1)

Supplementary material (online resources - J Cancer Res Clin Oncol)

Final analysis of the randomised PEAK trial: overall survival and tumour responses during first-line treatment with mFOLFOX6 plus either panitumumab or bevacizumab in patients with metastatic colorectal carcinoma

Fernando Rivera^1^ · Meinolf Karthaus^2^ · J Randolph Hecht^3^ · Isabel Sevilla^4^ · Frédéric Forget^5^ · Gianpiero Fasola^6^ · Jean‑Luc Canon^7^ · Xuesong Guan^8^ · Gaston Demonty^9^ · Lee S Schwartzberg^10^

^1^Hospital Universitario Marqués de Valdecilla, Santander, Spain

^2^Städtisches Klinikum München, Klinikum Neuperlach, Munich, Germany

^3^David Geffen School of Medicine at University of California Los Angeles, Los Angeles, CA, USA

^4^Virgen de la Victoria University Hospital, Malaga, Spain

^5^Centre Hospitalier de l’Ardenne, Libramont, Belgium

^6^University Hospital Santa Maria della Misericordia, Udine, Italy

^7^Grand Hôpital de Charleroi, Charleroi, Belgium

^8^Amgen Inc., Biostatistics, Thousand Oaks, CA, USA

^9^Amgen (Europe) GmbH, Medical Development – Oncology, Zug, Switzerland

^10^West Clinic, Memphis, TN, USA

Correspondence to: Dr Fernando Rivera, Hospital Universitario Marqués de Valdecilla, Av. de Valdecilla, 39008 Santander, Spain. Tel: +34 942202515; Fax: +34 942203362; E-mail: [oncrhf@humv.es](mailto:oncrhf@humv.es)

Table A1 Summary of progression-free survival, overall survival and objective response results (*RAS* wild-type/*BRAF* mutant population)

|  | ***RAS* wild-type/*BRAF* mutant** | |
| --- | --- | --- |
|  | **Panitumumab  + mFOLFOX6 (*n=*11)** | **Bevacizumab  + mFOLFOX6 (*n=*3)** |
| **Progression-free survival** |  |  |
| Patients with event, *n* (%) | 9 (82) | 3 (100) |
| Median, months (95% CI) | 7.4 (3.9, 10.7) | 9.5 (5.6, 16.6) |
| HR (95% CI) | 2.91 (0.36, 23.9) | |
| *p*-value^*^ | 0.32 | |
| **Overall survival** |  |  |
| Patients with event, *n* (%) | 9 (82) | 3 (100) |
| Median, months (95% CI) | 17.5 (9.1, 28.8) | 21.0 (5.6, 21.0) |
| HR (95% CI) | 0.66 (0.14, 3.21) | |
| *p*-value^*^ | 0.60 | |
| **Objective response** |  |  |
| Responders,^†^ *n* | 7 | 1 |
| ORR,^†^ % (95% CI) | 63.6 (30.8, 89.1) | 33.3 (0.8, 90.6) |
| Difference in rates, % (95% CI) | 30.3 (-32.6, 70.0) | |
| Odds ratio^‡^ (95% CI) | 1.8 (0.0, 156.7) | |
| *p*-value^¶^ | 1.00 | |

^*^From stratified Cox model. ^†^As assessed by RECIST. ^‡^Defined as the odds of having an objective response in the panitumumab + mFOLFOX6 arm relative to the odds in the bevacizumab + mFOLFOX6 arm adjusted for interactive voice response system (IVRS) randomisation factors. ^¶^From stratified exact test

*CI* confidence interval, *HR* hazard ratio, *ORR* objective response rate

Table A2 Progression-free and overall survival outcomes by tumour shrinkage at week 8 (*RAS* wild-type population – intra-treatment comparisons)

|  | **Tumour shrinkage at week 8** | | | | |
| --- | --- | --- | --- | --- | --- |
|  | **Panitumumab + mFOLFOX6** | | **Bevacizumab + mFOLFOX6** | | |
|  | **<30%** | **≥30%** | **<30%** | **≥30%** | |
| Patients with shrinkage, *n* (%) | 29 (36) | 51 (64) | 41 (55) | 33 (45) | |
| Median PFS, months (95% CI) | 11.6 (7.5, 15.4) | 13.0 (10.9, 18.1) | 9.7 (7.5, 12.9) | 11.1 (9.0, 16.6) | |
| HR (95% CI) | 0.62 (0.35, 1.10) | | 0.60 (0.36, 1.01) | | |
| Median OS, months (95% CI) | 34.2 (17.5, 42.3) | 43.8 (36.4, 63.0) | 23.9 (20.1, 29.0) | | 35.1 (29.9, NE) |
| HR (95% CI) | 0.40 (0.22, 0.72) | | 0.43 (0.24, 0.78) | | |
|  | **Tumour shrinkage at week 8** | | | | |
|  | **Panitumumab + mFOLFOX6** | | **Bevacizumab + mFOLFOX6** | | |
|  | **<20%** | **≥20%** | **<20%** | **≥20%** | |
| Patients with shrinkage, *n* (%) | 20 (25) | 60 (75) | 28 (38) | 46 (62) | |
| Median PFS, months (95% CI) | 9.8 (4.2, 15.4) | 13.1 (10.9, 16.2) | 9.5 (7.4, 12.7) | 11.3 (9.2, 13.6) | |
| HR (95% CI) | 0.46 (0.25, 0.86) | | 0.60 (0.36, 1.01) | | |
| Median OS, months (95% CI) | 21.2 (14.1, 41.2) | 43.4 (36.4, 55.4) | 21.8 (15.3, 28.9) | | 32.5 (27.7, 47.4) |
| HR (95% CI) | 0.38 (0.20, 0.69) | | 0.42 (0.24, 0.75) | | |

*CI* confidence interval, *HR* hazard ratio, *NE* not evaluable, *OS* overall survival, *PFS* progression-free survival

Table A3 Summary of adverse events (*RAS* wild-type population)

| ***n* (%)** | **Panitumumab  + mFOLFOX6 (*n=*86)** | **Bevacizumab  + mFOLFOX6 (*n=*80)** |
| --- | --- | --- |
| Any AE | 86 (100) | 80 (100) |
| Worst grade of 3 | 60 (70) | 43 (54) |
| Worst grade of 4 | 17 (20) | 15 (19) |
| Worst grade of 5 | 4 (5) | 7 (9) |
| Any serious AE | 37 (43) | 32 (40) |
| Any AE leading to permanent discontinuation of any study drug | 25 (29) | 24 (30) |
| Not serious | 19 (22) | 17 (21) |
| Serious | 6 (7) | 8 (10) |
| Worst grade 3+ and ≥5% difference between treatment groups |  |  |
| Rash | 13 (15) | 0 (0) |
| Hypomagnesaemia | 7 (8) | 0 (0) |
| Stomatitis | 6 (7) | 0 (0) |
| Decreased appetite | 5 (6) | 1 (1) |
| Dehydration | 5 (6) | 1 (1) |
| Acne | 4 (5) | 0 (0) |
| Dermatitis acneiform | 4 (5) | 0 (0) |
| Deep vein thrombosis | 2 (2) | 6 (8) |
| Hypertension | 0 (0) | 6 (8) |

*AE* adverse event

Fig. A1 Forest plot for overall survival in patient subpopulations (*RAS* wild-type population)


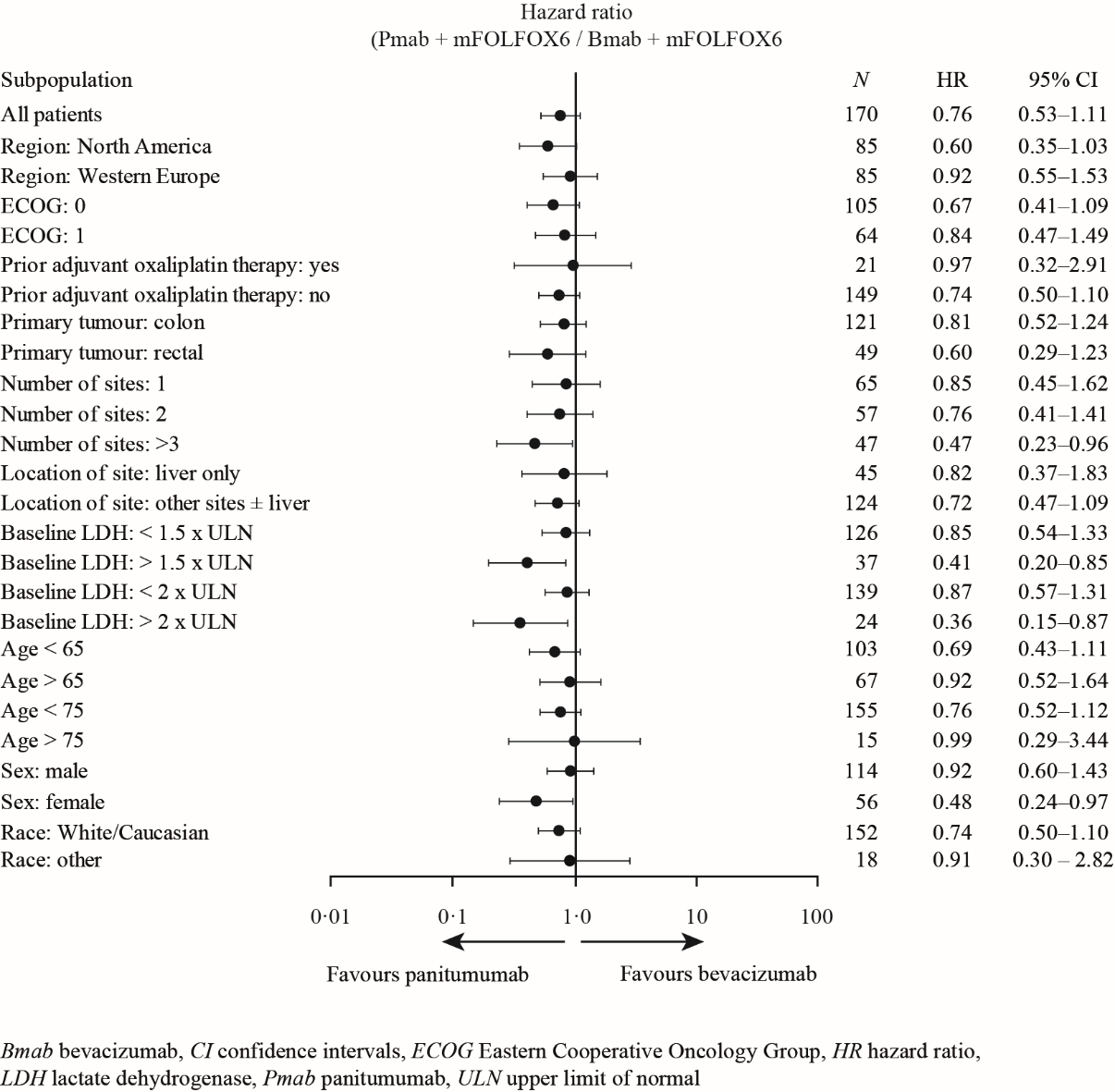


Fig. A2 Kaplan-Meier estimates of a) Progression-free survival and b) Overall survival (*RAS* wild-type/*BRAF* wild-type population)


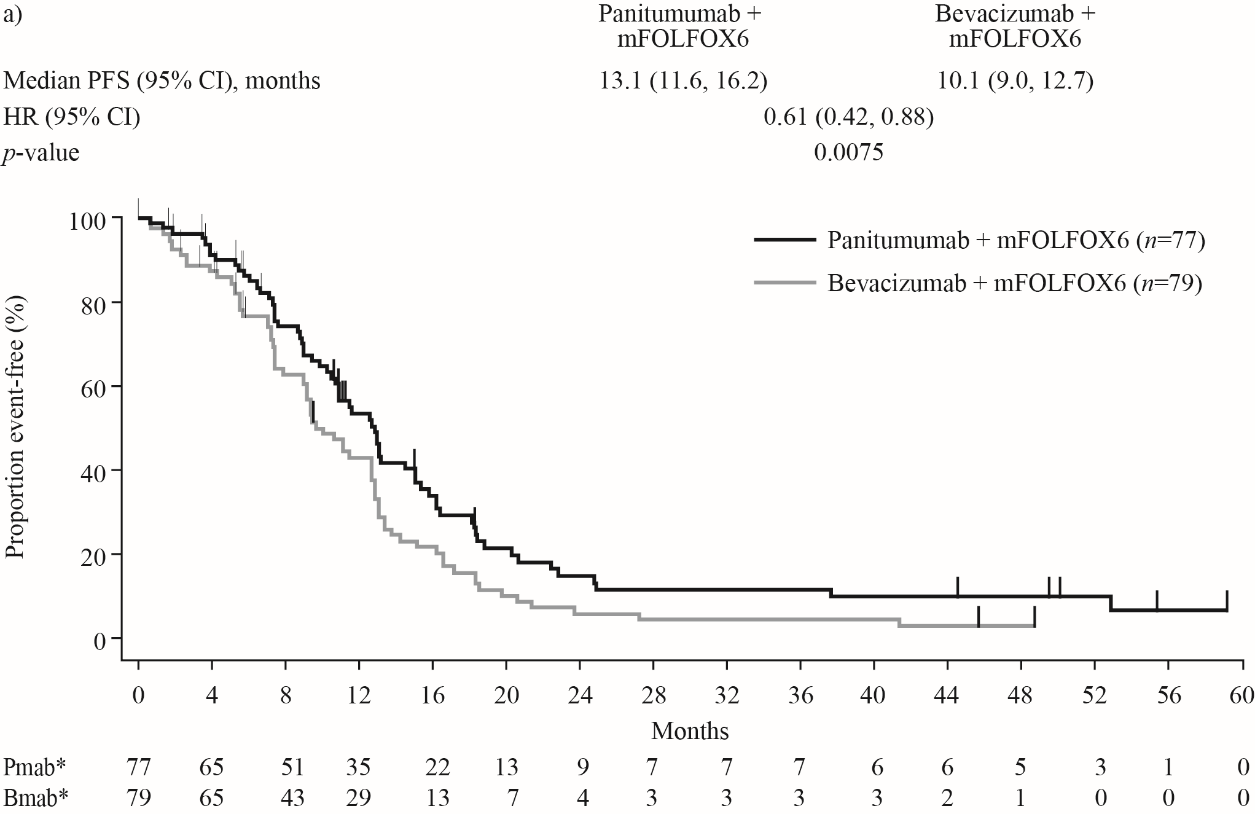


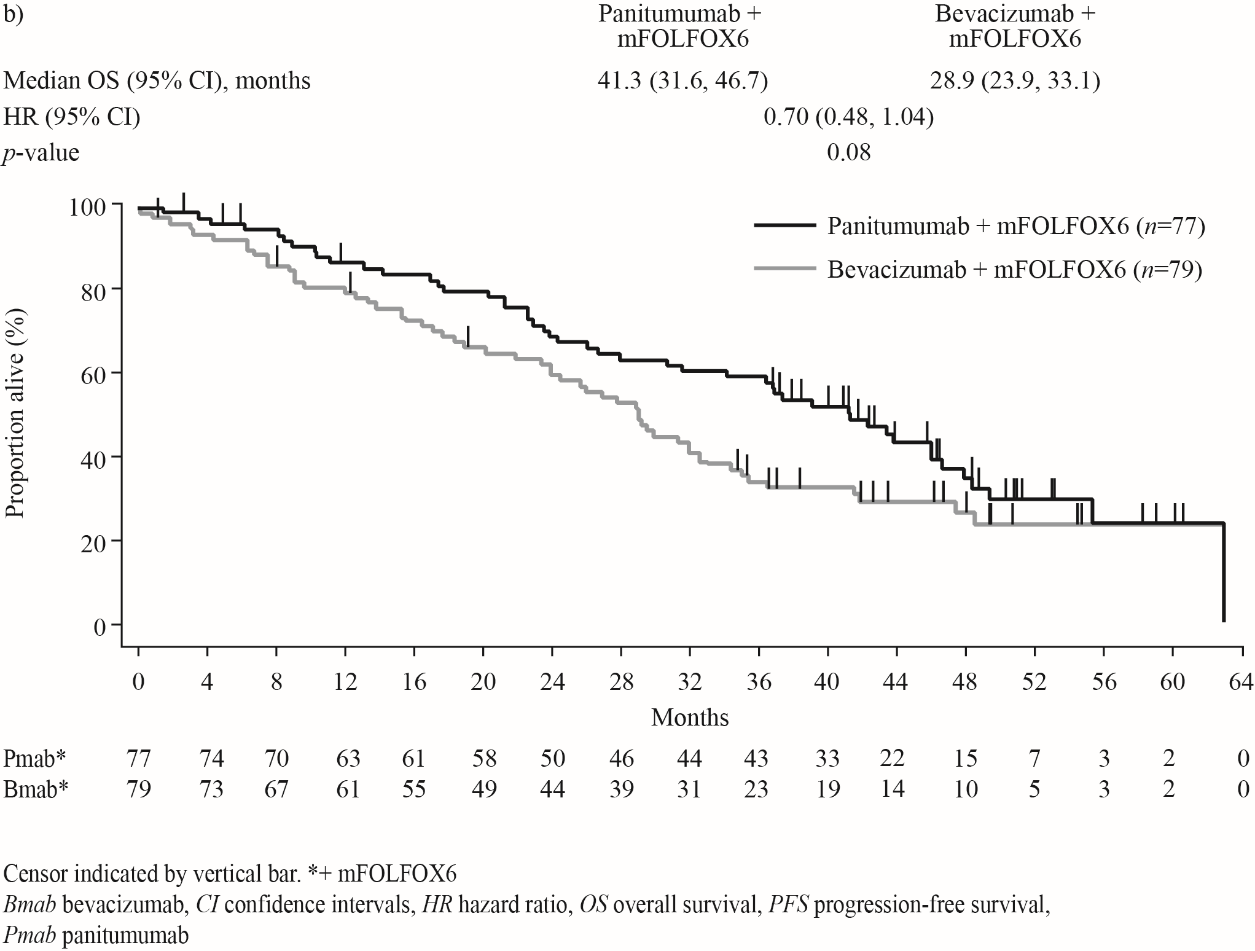


Fig. A3 Kaplan-Meier estimates of a) Progression-free survival and b) Overall survival in patients with tumour shrinkage of ≥30% at week 8 (*RAS* wild-type population)


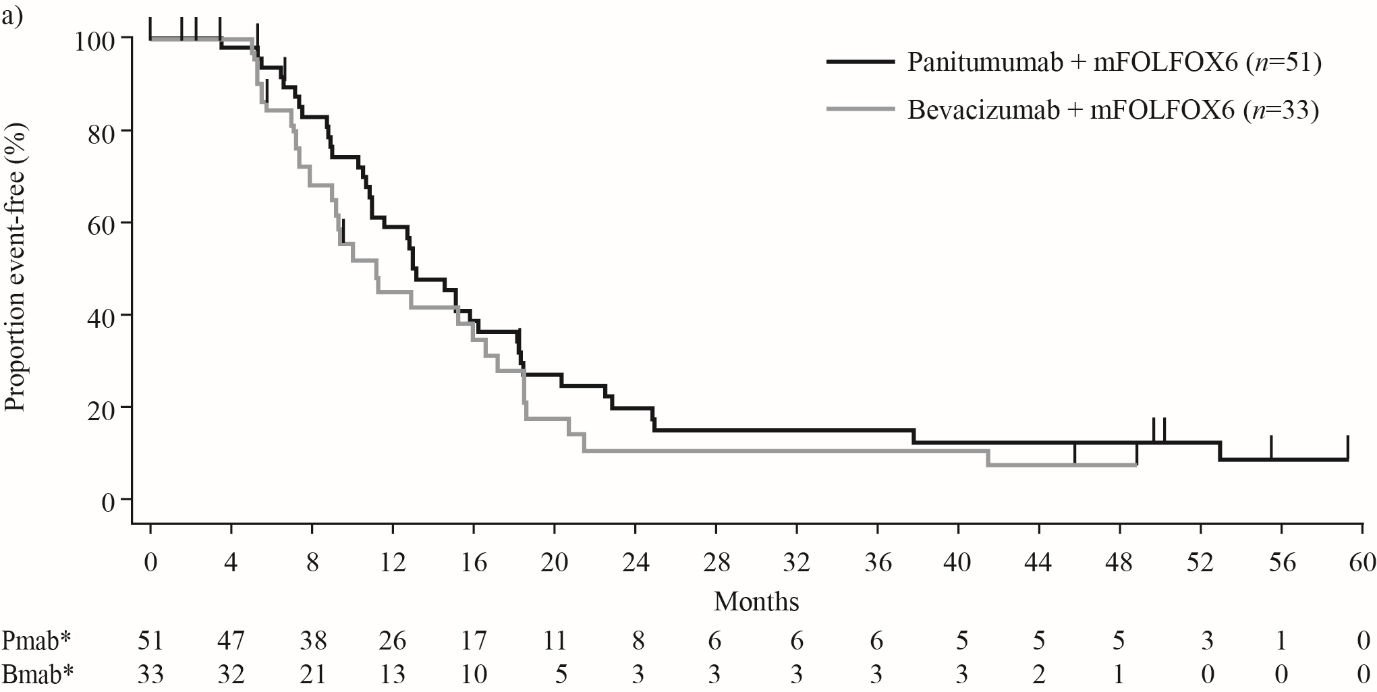


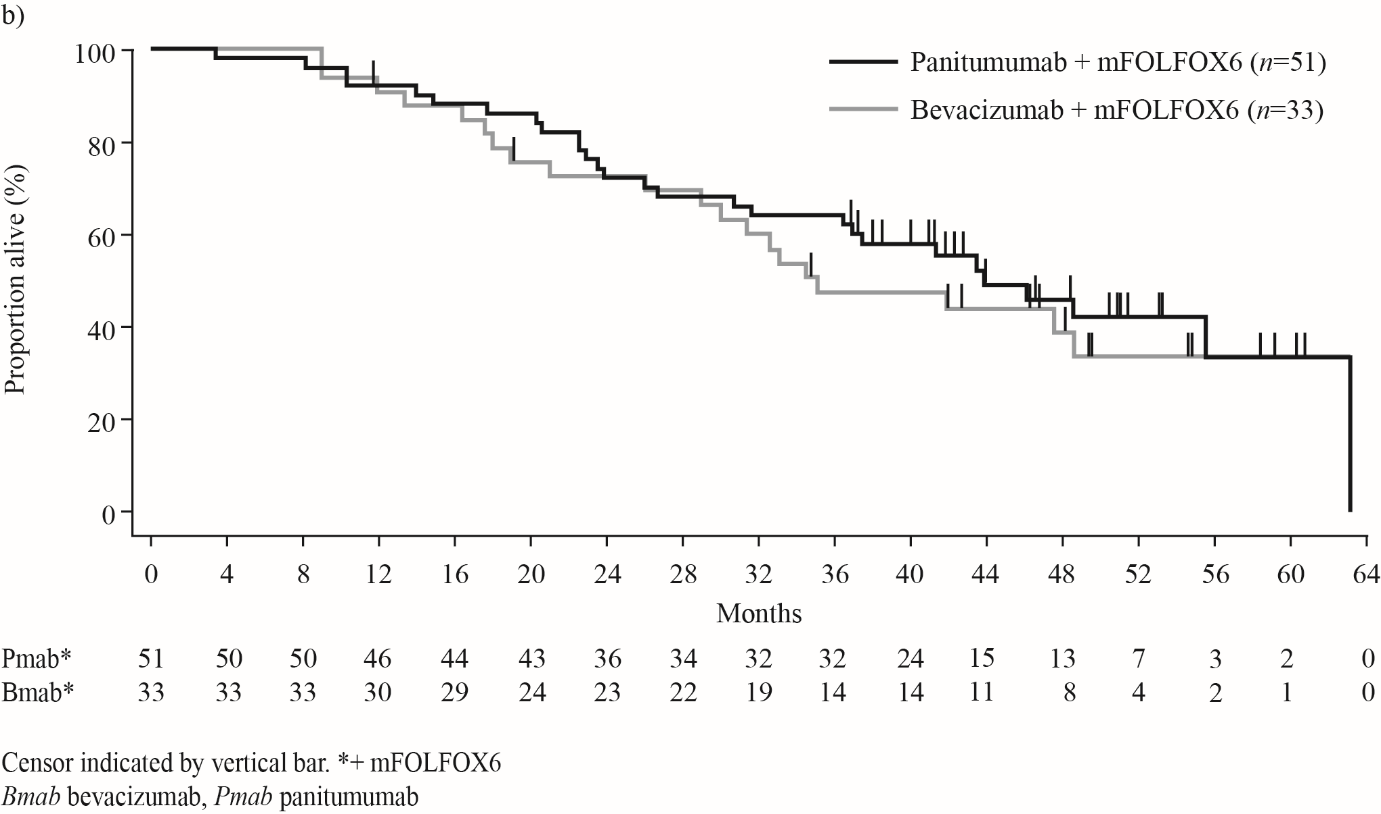

Supplement: Supplementary file 1 — (DOCX 1211 kb) [file 384_2017_2800_MOESM1_ESM.docx]
